# Development and initial validation of the eating disorder-related rumination assessment scale in a Turkish non-clinical sample

**DOI:** 10.1186/s40359-026-05457-7

**Published:** 2026-08-26

**Authors:** Dilan Gulacan, Caner Doğrusever, Sevgi Bektas-Dag, Guzin Mukaddes Sevincer

**Affiliations:** 1 Department of Psychology, Hacettepe University Faculty of Letters, 06800 Ankara, Türkiye; 2 Department of Psychological Counselling and Guidance, Faculty of Education, Siirt University, 56100 Siirt, Türkiye; 3 Department of Psychiatry, Prof. Dr. Cemil Taşçıoğlu City Hospital, 34384 Istanbul, Türkiye

**Keywords:** Eating disorders, Eating pathology, Rumination, Ruminative thinking, Psychometric validation, Scale development

## Abstract

**Objectives:**

There is no valid measurement tool available in Türkiye to assess rumination specific to eating disorders. This study is based on the assumption that conceptualizing rumination within the framework of Goal Progress Theory will enable a comprehensive assessment of goal-related and control-focused cognitive processes, which are characteristic features of eating disorders. Accordingly, the research was conducted to develop and examine the psychometric properties of the Eating Disorder-Related Rumination Assessment Scale (ED-RRA) in a Turkish adult sample.

**Methods:**

A total of 779 Turkish adult participants (186 men, 593 women) completed the final 12-item ED-RRA. Psychometric analyses included exploratory and confirmatory factor analysis (EFA, *n* = 392; CFA, *n* = 387), both estimated with a robust categorical estimator (WLSMV), as well as internal consistency, test-retest reliability, and convergent validity. External validation measures assessing depression (PHQ-9), anxiety (GAD-7), general rumination (RRS), and eating disorder symptoms (EDE-Q) were administered only in the CFA sample (*n* = 387).

**Results:**

Factor analyses supported a single factor. Internal consistency was excellent in both samples (Cronbach’s α = 0.93–0.94; McDonald’s ω = 0.93–0.94), and temporal stability over a 15-day interval was good (ICC = 0.88). The ED-RRA demonstrated strong convergent validity with measures for ED pathology (*r* = 0.76). The ED-RRA showed small-to-moderate correlations with the PHQ-9, GAD-7, and RRS (*r* = 0.34–0.37). In a hierarchical regression, the ED-RRA predicted ED pathology beyond general rumination, depression, and anxiety (ΔR² = 0.44), although this increment partly reflects content shared with the EDE-Q.

**Discussion:**

The ED-RRA showed promising preliminary psychometric properties in a non-clinical Turkish adult sample. In contrast to existing measures, it was constructed to target goal-related and control-focused ruminative content. As the present evidence is preliminary and confined to a community sample, the scale is not yet intended for clinical or diagnostic use.

**Supplementary Information:**

The online version contains supplementary material available at https://doi.org/10.1186/s40359-026-05457-7.

## Introduction

Rumination is a cognitive process in which a person passively and repetitively focuses attention on negative experiences—such as life events and emotions—and their possible meanings, causes, and consequences [1]. Despite extensive research, no consensus exists on a single definition and standardized measurement approach for rumination, as its conceptualization varies across theoretical frameworks [2]. Nevertheless, it is widely accepted that rumination functions as a transdiagnostic risk factor implicated in various psychiatric disorders, including eating disorders (EDs) [3, 4].

Recent studies indicate that individuals with EDs exhibit higher levels of rumination compared to the general population [5, 6]. Furthermore, evidence suggests a reciprocal relationship between rumination and ED symptoms [4]. Rumination thus serves as an important cognitive risk factor in ED psychopathology, contributing to the development and persistence of symptoms including ED-related fears, avoidance and safety behaviours, and cognitive rigidity [7–9]. This process can worsen psychopathology by intensifying negative mood states and reinforcing maladaptive thinking patterns, impairing problem-solving abilities, reducing engagement in goal-directed activities, and diminishing sensitivity to environmental feedback [10]. In individuals with EDs, chronic rumination may further deplete motivational resources, lower stress tolerance, and reduce social support, thereby maintaining or exacerbating the disorder over time [10–12]. Given the importance of rumination in developing and maintaining ED psychopathology, accurately assessing this cognitive process is essential.

Over the past decade, research has increasingly focused on disorder-specific rumination—repetitive thoughts centred on particular psychopathologies—to improve treatment planning, primary prevention, and basic research [13, 14]. In the context of EDs, Cowdrey and Park [15] developed the Ruminative Response Scale for Eating Disorders (RRS-ED), a self-report screening tool designed to assess the severity of rumination specific to EDs, in a healthy sample. RRS-ED was adapted from a ten-item subset of the Ruminative Response Scale (RRS; [16]), retaining its two core subdimensions: reflection and brooding. Reflection refers to a purposeful and analytical form of self-focused cognition aimed at understanding and evaluating one’s emotional states and behavioural responses, whereas brooding involves a passive, self-critical, and perseverative pattern of repetitive negative thinking [16].

Minimal modifications were made to the RRS to tailor it to the ED context. For example, in the brooding subdimension, the item “Think ‘Why do I have problems that other people do not have?’” was revised to “Think ‘Why do I have problems controlling my eating, weight, and/or body shape when other people do not?’.” Similarly, in the reflection subdimension, the item “Go someplace alone to think about your feelings” was adapted to “Go someplace alone to think about your feelings regarding eating, weight, and/or body shape,” [15]. Psychometric analyses revealed that the RRS-ED comprises two factors with a total of nine items and demonstrates acceptable internal consistency (Cronbach’s alpha for brooding = 0.89, for reflection = 0.63). Despite these promising results, evidence regarding the cross-cultural validity of the RRS-ED’s factor structure remains limited. Marques et al. found that, in a Portuguese community sample, a general rumination factor provided a better fit than the original brooding and reflection subscales [17].

Although the RRS-ED has been utilized as a valuable tool for assessing rumination specific to EDs, it presents some limitations. Specifically, it is adapted from the RRS, which was originally developed to assess the severity of depressive rumination within the framework of the Response Styles Theory [18]. As such, the RRS-ED builds upon the RRS’s conceptualization of rumination as a repetitive and passive response to negative mood, which intensifies and prolongs negative emotional states [18]. However, as outlined below, alternative theoretical models offer more nuanced perspectives on the mechanisms underlying rumination.

Goal Progress Theory [19] suggests that rumination arises not solely from negative affect but from the perceived failure to make progress toward personally significant goals. While individuals pursue many goals in daily life, ruminative processes are specifically linked to higher-order goals that carry personal significance. Given the perfectionistic expectations, rigid goals, and intense control efforts commonly observed in EDs, this perspective is particularly relevant [20]. In EDs, perfectionism often centres on achieving an ideal weight, body shape, and eating behaviour. Current research distinguishes between two types of perfectionism: self-oriented perfectionism (SOP), which involves setting unrealistically high standards for oneself, and socially prescribed perfectionism (SPP), which reflects the belief that others hold excessively high expectations and that one must meet these to avoid potential criticism [21]. SPP, in particular, is associated with a fear of negative evaluation and the perception that others expect them to be flawless and that they will be subjected to intense criticism if they fail to meet these expectations. Empirical findings consistently highlight the prominence of perfectionistic tendencies in EDs, linking them to interpersonal difficulties and ruminative responses [22–24]. Although individuals set numerous goals in daily life, they do not engage in ruminative processes for every unfulfilled goal. According to Goal Progress Theory, rumination is particularly associated with higher-order goals that hold personal significance [19]. To test this hypothesis, Lavallee and Campbell [25] asked participants to identify their higher-order goals and to record salient daily events, moods, and cognitive–emotional responses related to these events over a two-week period. Their findings showed that negative events connected to higher-order goals, compared to unrelated negative events, were linked to greater negative affect, increased self-focused attention, heightened self-concept confusion, and elevated rumination. Within this framework, behaviours and thoughts such as calorie counting, meal planning, body comparisons, and concerns about loss of control can be understood as important components of the rumination cycle. Goal Progress Theory also posits that goal failures leave a lasting imprint on the mind [26]. Information related to unattainable goals becomes more accessible within the individual’s cognitive system, which in turn causes attention to be automatically drawn to this information, thereby fuelling ruminative thought cycles [19]. Carver and Scheier’s control process model further supports this, proposing that individuals continuously compare their current state against their goals through a feedback loop, with perceived insufficient progress generating negative affect and cognitive preoccupation [27]. In addition to the goal-discrepancy processes emphasized, behavioural avoidance and thought suppression also play crucial roles in ED-specific rumination. For example, Ironic Processes Theory [28] highlights that deliberately trying to suppress certain thoughts or behaviours can backfire cognitively, resulting in heightened rumination on exactly what is being suppressed. Thus, it is important to consider thought content that reflects the mental costs of the intense control efforts made by individuals with EDs to achieve their goals, such as the persistent preoccupation with food observed among those engaged in long-term dietary restriction [29].

Taken together, these models offer alternative frameworks. While the RRS-ED offers a valuable starting point, its foundation in mood-based conceptualizations limits its capacity to sufficiently capture aspects of rumination involving self-oriented and socially prescribed perfectionistic expectations, intense control efforts, and perceived failures in achieving personally meaningful goals. These themes of ruminative thought content may warrant more targeted assessment.

Guided by this theoretical framework, item generation in the present study was organized around three core content domains derived from the goal-focused cognitive literature on EDs. The central criterion was whether each item reflected repetitive and involuntary cognitive preoccupation arising from perceived insufficient progress toward personally meaningful goals related to eating behaviour, body shape, or weight.

The first domain covers repetitive and involuntary thoughts associated with perceived failure to meet personal standards regarding body weight, shape, and eating behaviour in the context of self-oriented perfectionism. Cognitive representations of unattained goals remain mentally active and fuel involuntary repetitive thinking [26]. Items in this domain were developed to reflect ruminative cognitions directed at unattained body and weight goals. Sample items: “Thoughts like ‘I wish I hadn’t eaten so much’ and ‘I wish I was at the weight I want to be’ occupy my mind” and “Despite trying not to think about it, thoughts of failing to achieve my body shape, weight, or eating goals keep coming back to my mind.”

The second domain covers repetitive preoccupation with the perceived impact of body shape and weight on interpersonal relationships and social approval, in the context of socially prescribed perfectionism. This reflects perceived insufficient progress toward social approval goals and sustains rumination around interpersonal failure [19]. Sample items: “I cannot get rid of the idea that problems with my body shape, weight, or eating behaviours affect my social life” and “I cannot get rid of the idea that problems with my body shape, weight, or eating behaviours affect my romantic relationships.”

The third and most extensive domain covers repetitive cognitive preoccupation related to control efforts and goal-monitoring processes. These reflect a feedback loop that continuously monitors the gap between current state and desired goals, consistent with Carver and Scheier’s control process model [27]. This domain also incorporates the paradoxical cognitive cost described by Ironic Processes Theory [28]: attempts to suppress thoughts about restricted foods increase their intrusive frequency [29]. Sample items: “I spend most of the day thinking about how to burn off all the calories I ate (e.g., should I exercise or use laxatives),” “I spend most of the day thinking about my meals (e.g., what to eat, how much to eat, when to eat, and how many meals I should have?),” and “When trying to control my body shape, weight, or eating behaviours, I cannot stop myself from thinking about the foods I enjoy.”

### Aim of the current study

To date, no scale assessing eating disorder-specific rumination has been adapted or developed for use in Türkiye. While adapting an existing measure such as the RRS-ED was considered, certain conceptual and structural aspects of the scale suggested that developing a new instrument might be more appropriate for capturing the specific features emphasized in contemporary cognitive models of eating disorders. The rationale for developing a new instrument rather than adapting the RRS-ED was conceptual rather than a claim of empirical superiority. In other words, our aim was to reorient the assessment toward the goal-related and control-focused processes emphasised by contemporary cognitive models, not to assert that the ED-RRA outperforms the RRS-ED, a claim that the present design cannot support. Accordingly, the present study aims to develop and validate the Eating Disorder-Related Rumination Assessment Scale (ED-RRA) in a Turkish adult population.

## Methods

### Procedure

**Fig. 1:**
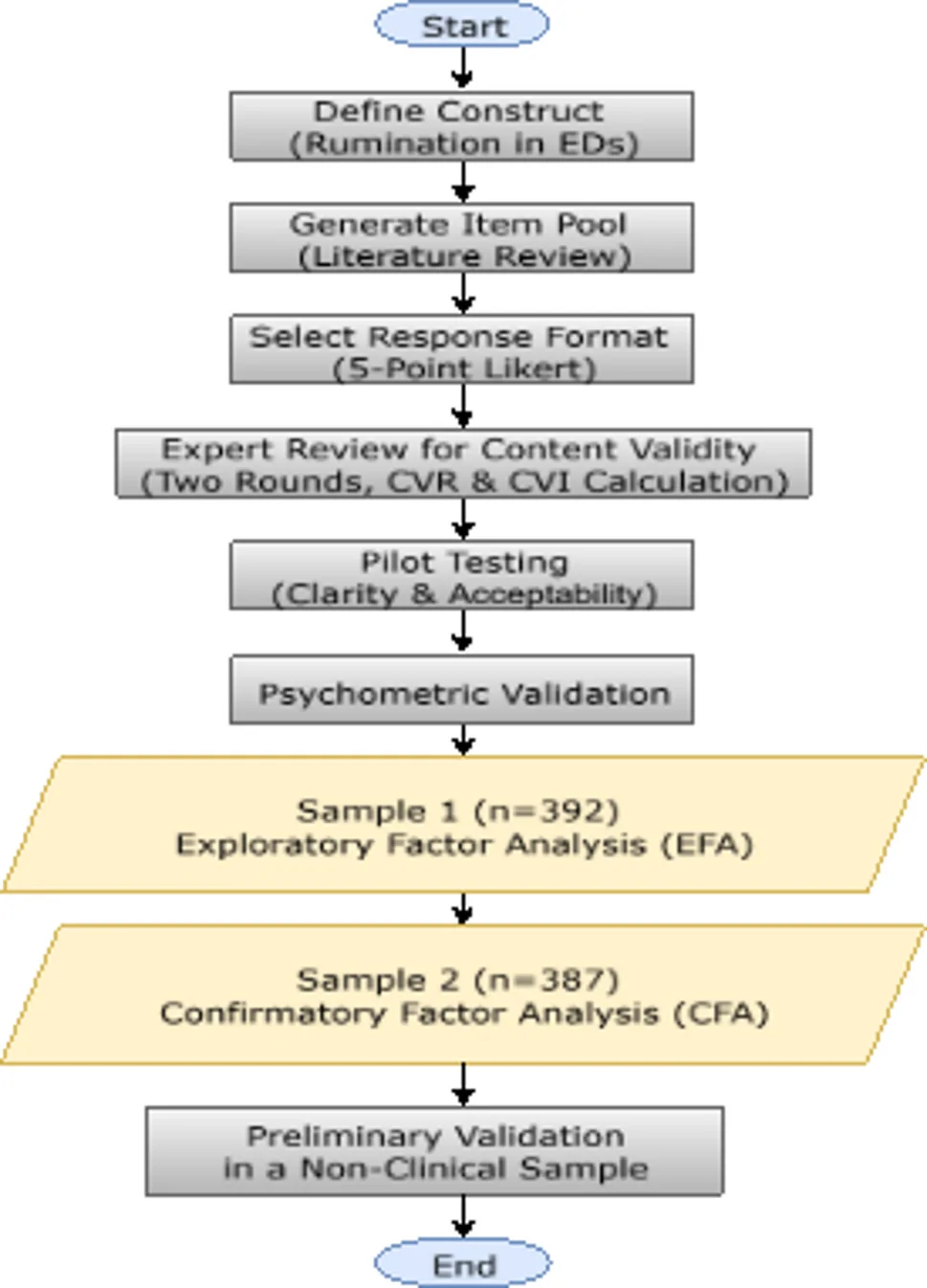
A schematic representation of the scale development and preliminary validation process

The overall scale development process, guided by the framework proposed by DeVellis and Thorpe’s [30], is visually summarized in Fig. 1.

### Phase I – clearly define the concept to be measured

Our aim was to examine the theoretical structure of rumination in depth and to evaluate its relevance to eating disorders (EDs). To achieve this, a literature review was conducted on 15 December 2022 using three electronic databases: PubMed, Scopus, and Web of Science. The following search terms were used, applied to titles, abstracts, and keywords: (“Eating Disorder” OR “Eating Pathology” OR “Anorexia” OR “Bulimia” OR “Binge Eating” OR “Body Image” OR “Body Dissatisfaction”) AND (“Rumination” OR “Ruminative Thinking” OR “Repetitive Thinking” OR “Rumination Theory” OR “Rumination Theoretical Model”).

Studies were excluded if (a) full-text access was not available or (b) they were not published in English or Turkish. The titles and abstracts of all retrieved studies were screened according to these eligibility criteria, and the full texts of the selected articles were subsequently reviewed in detail by the authors. Key themes and constructs relevant to the development of a theory-driven measurement tool were identified and used to inform the item generation phase of the scale development process.

Several inclusion criteria guided the development of the initial item pool. Specifically, items were required to: (1) reflect cognitive preoccupation arising from perceived insufficient progress toward personally meaningful higher-order goals related to eating behaviour, body shape, or weight; (2) represent a repetitive, involuntary, and persistent cognitive process; (3) capture recurrent cognitive preoccupation with unattained goals in the context of self-oriented or socially prescribed perfectionistic expectations, or the intensive monitoring and control efforts directed toward attaining such goals; and (4) reflect cognitive patterns commonly observed in both clinical and non-clinical manifestations of eating disorder psychopathology.

Exclusion criteria included: (1) statements that did not capture the defining characteristics of rumination, such as repetitiveness, involuntariness, and perceived lack of control; (2) statements showing substantial conceptual overlap with constructs theoretically distinct from rumination, including future-oriented threat anticipation characteristic of anxiety, ego-dystonic obsessive content, or processes resembling constructive problem solving; and (3) overly specific statements pertaining to particular clinical subgroups or expressions that could limit the conceptual generalizability of the scale.

### Phase II – generation of an item pool

The initial item pool was developed based on findings obtained from the literature review on rumination in the context of eating disorders. Through a comprehensive analysis of conceptual models and empirical findings, the core content domains of ED-specific rumination were identified: repetitive and involuntary preoccupation with unattained goals related to body weight, shape, and eating behaviour; recurrent thoughts about perceived failure and inadequacy in the context of self-oriented and socially prescribed perfectionism; and sustained cognitive preoccupation with goal-monitoring processes including calorie counting, meal planning, and body checking. Guided by these theoretical insights, the authors generated an initial pool of 52 items aimed at capturing ED-specific rumination.

### Phase III – determine the format for measurement

The response format for the ED-RRA was designed as a five-point Likert scale, with response options ranging from “Never” to “Always” (Never, Rarely, Sometimes, Often, Always). The format was selected due to its widespread application in psychological research and its utility in capturing the frequency and intensity of cognitive and behavioural processes [30].

### Phase IV – expert review of the initial item pool

An expert panel consisting of seven professionals specializing in EDs—three psychiatrists, two psychologists, and two dietitians—was assembled to evaluate the initial 52-item pool. Using a structured evaluation form based on predefined criteria, panel members independently assessed each item in terms of content relevance (i.e., the extent to which the item reflected ED-specific rumination), theoretical alignment (i.e., consistency with the conceptual framework guiding scale development, including Goal Progress Theory and related models of cognitive control and thought suppression), and clarity (i.e., the comprehensibility and precision of item wording). During this review, items containing highly specific or narrowly defined wording (e.g., “my cheeks,” “my legs,” “a flat stomach,” “calorie restriction,” or “vomiting”) were reformulated using broader and more inclusive expressions such as “body shape, weight, or eating behaviours.” The purpose of these revisions was to ensure that items reflected the broader cognitive themes of ED-specific rumination rather than symptoms specific to particular ED subgroups, thereby enhancing the applicability of the scale across both clinical and non-clinical populations. Items judged to be inadequate in terms of both content relevance and theoretical alignment were removed without revision. This process reduced the item pool from 52 to 34 items.

In the second stage, the 34-item pool underwent a more comprehensive content validity evaluation. Each item was rated by the expert panel for essentiality, and the Content Validity Ratio (CVR) [31] was calculated for all 34 items. Based on the critical value recommended by Ayre and Scally for a seven-member panel, a CVR of ≥ 0.99, corresponding to unanimous “essential” ratings, was used as the quantitative criterion for item retention [32]. No item below this threshold was retained, and no item meeting the threshold was removed. For items falling below the threshold, the qualitative comments provided by the experts were reviewed to identify the conceptual basis for their ratings, including concerns regarding the defining characteristics of ruminative thinking, conceptual overlap, theoretical relevance, and parsimony. These qualitative evaluations provided the conceptual rationale for item removal and also informed refinement of the final scale; where appropriate, specific content from removed items was incorporated into corresponding retained items as parenthetical examples. This process resulted in the final 12-item version of the scale. Complete item-level expert ratings, CVR values, retention/removal decisions, and qualitative rationales are presented in Supplementary Table S3. In addition, the Content Validity Index (CVI) was calculated to assess overall agreement among experts, yielding a scale-level CVI of 1.00.

### Phase V – administration of items to a development sample

A pilot study was conducted to evaluate the clarity, comprehensibility, acceptability, and ease of response of items in the preliminary scale. This procedure focused on participants’ evaluative judgments about the items rather than their cognitive response processes and did not involve a formal cognitive-interviewing protocol, such as think-aloud administration or systematic verbal probing. A sample size of at least 10 participants is recommended as sufficient for evaluating the clarity of instructions, item wording, format acceptability, and ease of use in a pilot study [33]. This study used a non-clinical sample of 20 university students (12 female, 8 male). Participants completed the provisional version of the ED-RRA and subsequently provided structured feedback on item wording, clarity of instructions, format acceptability, and ease of response. The results indicated that no substantive revisions were necessary. Participants completed the scale within an average of 5–7 min, and their feedback affirmed the clarity, coherence, and ease of administration of the instrument—supporting its appropriateness for broader implementation in subsequent validation phases. Although the pilot was conducted with a non-clinical sample within the normal BMI range, prior research suggests that subclinical eating disorder symptoms frequently approach clinical severity and that core cognitive-behavioural features are commonly observed in community samples [34–36].

### Phase VI – evaluation of items

The validity and reliability of the ED-RRA were examined through a two-stage analytical approach using two independent samples (*N* = 779), given that CFA goodness-of-fit tests are considered insignificant when conducted on the same dataset initially used for EFA exploration. To explore the scale’s underlying structure, EFA was conducted on the first sample. CFA was then conducted on the second independent sample to verify the factor structure identified in the EFA. Both sample sizes met the recommended thresholds for psychometric analysis and scale development [37, 38]. Further details are provided in the Results.

### Phase VII – scale length optimization

As described in Phase IV, items failing to meet the quantitative content-validity criterion were removed following review of their qualitative rationales, including conceptual overlap, theoretical misalignment, overly specific content, and other departures from the intended ruminative construct, resulting in a 12-item version of the ED-RRA. In the present phase, EFA was conducted to empirically evaluate whether this item set adequately represented the underlying construct. EFA showed that all 12 items had high item-total correlations and factor loadings, supporting a unidimensional structure (as shown in the Results). The finalized 12-item ED-RRA is presented in Supplementary Material Table S1 in Turkish, together with English translations provided solely to facilitate reporting and interpretation of the item content for English-speaking readers. These translations were prepared after the Turkish scale had been finalized and were not part of the scale development, adaptation or psychometric evaluation process. The finalized Turkish items were translated into English by two clinical psychologists and independently back-translated into Turkish by a bilingual language specialist who had not seen the original items. Discrepancies between the translations were reviewed and resolved to establish the final English wording. The final English form has not undergone psychometric evaluation and should not be used for assessment without formal validation.

### Main study: psychometric validation of the ED-RRA

### Participants

Participants were Turkish native speakers recruited through voluntary participation using a snowball sampling strategy [39]. The exclusion criteria for participants were as follows: (1) a history of chronic medical conditions, (2) a history of psychological disorders, (3) a current diagnosis of an eating disorder, and (4) being pregnant or breastfeeding at the time of the study. These exclusion criteria were applied for distinct reasons. A current eating-disorder diagnosis was treated as an exclusion because the present study was conceived as a community-based initial validation, the goal of which was to be the establishment of the instrument’s psychometric foundations prior to clinical validation. A history of chronic medical conditions, as well as pregnancy and breastfeeding, were excluded because such states may physiologically alter appetite, weight, and body perception and thereby confound the target construct. A self-reported history of diagnosed psychological disorders was excluded to limit the confounding influence of diagnosed comorbidity; importantly, this criterion did not remove symptom-level variability, as evidenced by the substantial range of depressive and anxiety symptoms observed across the sample (see Table 1). All participants provided self-reported information regarding inclusion and exclusion criteria. Both recruitments were conducted online through a web-based questionnaire (Google Form, forms.google.com), and participants did not receive any form of payment. Informed consent was obtained from all participants using an approved participant information sheet and consent form. Procedures were carried out in accordance with the principles of the Declaration of Helsinki and were approved by the Siirt University Ethics Committee (Reference No: 440/08.08.2022).

**Table 1:** Descriptors of the samples recruited for the EFA and CFA

	Sample 1 (*n* = 392)	Sample 2 (*n* = 387)
***Sociodemographic characteristics***	**M ± SD or ** ***n*** ** (%)**	
Age	27.47 ± 8.49	24.21 ± 6.08
BMI	23.48 ± 3.87	22.68 ± 7.48
Gender		
Female	286 (73.0%)	307 (79.3%)
Male	106 (27.0%)	80 (20.7%)
***Measures***	**M ± SD**	
ED-RRA	24.55 ± 10.53	25.39 ± 10.02
RRS	NA	22.40 ± 7.49
EDE-Q Global	NA	1.17 ± 1.10
EDE-Q Restraint	NA	1.00 ± 1.28
EDE-Q Eating Concern	NA	0.77 ± 1.04
EDE-Q Shape Concern	NA	1.52 ± 1.38
EDE-Q Weight Concern	NA	1.39 ± 1.28
PHQ-9	NA	10.86 ± 6.01
GAD-7	NA	8.60 ± 5.93

Notes: BMI = Body Mass Index, CFA = Confirmatory Factor Analysis, EDE-Q = Eating Disorders Examination Questionnaire, EFA = Exploratory Factor Analysis, GAD-7 = Generalized Anxiety Disorder Questionnaire, M = Mean, NA = Not Applicable, PHQ = Patient Health Questionnaire, ED-RRA = Eating Disorder-Related Rumination Assessment Scale, RRS = Ruminative Responses Scale, SD = Standard Deviation

### Measures

Beyond ED-RRA, all the measures used in the present study were validated in the Turkish context. For each measure, Cronbach’s alpha and McDonald’s omega were estimated.

#### Socio-demographics

The demographic questionnaire measured the following variables: age, gender, and history of psychiatric diagnosis. Self-reported weight and height details were also recorded, which were used to calculate body mass index (BMI) in kg/m^2^.

#### Eating disorders examination questionnaire (EDE-Q)

The 28-item EDE-Q was used to assess eating disorders [40]. Yucel et al. adapted the scale to Turkish and assessed its validity and reliability [41]. A global score is calculated by mean of the four subscale (Restraint, Eating Concern, Shape Concern, and Weight Concern) scores (range 0–6), consisting of 22 items scored on a 7-point Likert scale. Higher scores indicate greater levels of symptomatology. In the present study, the internal reliability for the scale was α = 0.94, and McDonald’s ω was 0.94.

#### Patient health questionnaire (PHQ-9)

The 9-item PHQ is a psychological test used to screen for and measure the severity of depression [42]. Güleç et al. [43] adapted to Turkish and conducted a validity and reliability study of this scale. Scores obtained from the scale range from 0 to 27. A higher score indicates greater depression. In the present study, the internal reliability for the scale was α = 0.87, and McDonald’s ω was 0.87.

#### Generalized anxiety disorder questionnaire (GAD-7)

The 7-item GAD is a short-form questionnaire used to screen for symptoms of generalized anxiety disorder and assess its severity [44]. Güleç et al. adapted to Turkish and conducted a validity and reliability study of this scale [43]. Scores obtained from the scale range from 0 to 21. A higher score indicates greater anxiety. In the present study, the internal reliability for the scale was α = 0.93, and McDonald’s ω was 0.93.

#### Ruminative responses scale (RRS)

The 10-item RRS was used to assess an individual’s tendency to ruminate when in a sad or depressed mood [16]. Erdur et al. adapted to Turkish and conducted the validity and reliability of this scale [45]. The scale comprises two subscales: brooding and reflection. Total scores range from 10 to 40, with higher scores indicating greater levels of brooding and reflection. In the present study, internal reliabilities were α = 0.84 and McDonald’s ω = 0.84 for brooding, α = 0.81 and McDonald’s ω = 0.82 for reflection, and α = 0.90 and McDonald’s ω = 0.91 for the total scale.

### Statistical analysis

Data analyses were performed using IBM SPSS Statistics 27.0, Mplus 8.3, as well as R version 4.6 with the psych package [46]. SPSS was used for descriptive statistics, item analysis, and reliability estimation; Mplus was used for the exploratory and confirmatory factor analyses with WLSMV estimation; and R was used for the polychoric parallel analysis as well as the assessment of multivariate normality. Categorical variables were expressed as percentages (%), while continuous variables were expressed as mean ± standard deviation (SD). The *p* < .05 threshold of significance was utilized for all analyses.

As described above, content validity in this study was systematically evaluated through a two-round expert review process. First, Content Validity Ratios (CVR) were calculated using the formula CVR = (ne - N/2)/(N/2), where “ne” represents the number of experts rating an item as essential and N is the total number of experts [31]. Based on Ayre and Scally’s [32] revised calculations for seven experts, a critical CVR threshold of ≥ 0.99 was applied as the quantitative benchmark for item essentiality. Following this, the Content Validity Index (CVI) was computed to assess overall agreement among experts. Experts rated each item on a 3-point scale: 1 (not essential), 2 (useful but not essential), and 3 (essential). The critical CVR value served as the operative criterion for item retention, and the panel’s qualitative evaluations of conceptual overlap, theoretical relevance, and parsimony provided the conceptual rationale for the removal of each below-threshold item (Supplementary Table S3).

Before running Exploratory Factor Analysis (EFA), an item analysis was conducted to evaluate the quality of the items. This process included calculating item-total score correlations to identify and remove poorly performing items. Although there is no specific standard as to the range within which the item-total correlation coefficient should be, Field [47] and De Vaus [48] suggest that the reliability power of the item is high if this coefficient ranges from 0.30 to 0.70.

Prior to the factor analyses, the distributional characteristics of the items were examined to inform the choice of estimator. Specifically, item-level skewness and floor frequencies, along with Mardia’s tests of multivariate skewness and kurtosis, were assessed. For the multivariate tests, the coefficients are reported alongside their associated test statistics, as the two quantities are distinct. Because the ED-RRA items employ a five-point ordinal response format and given the evidence of item non-normality reported in the Results, the items were treated as ordinal indicators rather than as continuous variables. Accordingly, both the exploratory and the confirmatory analyses were estimated from a polychoric correlation matrix using the robust weighted least squares estimator (WLSMV), which does not assume multivariate normality and is appropriate for categorical items [49, 50].

In Sample 1, the Kaiser-Meyer-Olkin (KMO) measure and Bartlett’s test of sphericity were first computed to assess the suitability of the data for factor analysis [37]. The number of factors to retain was then determined by considering the eigenvalue-greater-than-one criterion, the scree plot, the proportion of variance explained by the first factor, as well as a parallel analysis based on the polychoric correlation matrix. In this procedure, the eigenvalues obtained from the sample were compared with those generated from 500 random data sets, and a factor was retained when its observed eigenvalue exceeded the 95th percentile of the corresponding random eigenvalue. As the scale was designed to measure a single construct, no rotation was applied to the retained solution [51–53].

In Sample 2, the fit of the single-factor structure was assessed through CFA with WLSMV estimation. Model fit was evaluated using the scaled chi-square statistic together with its degrees of freedom, the comparative fit index (CFI) and the Tucker-Lewis index (TLI) (≥ 0.90), the standardized root mean square residual (SRMR ≤ 0.08), as well as the root mean square error of approximation (RMSEA ≤ 0.08) reported together with its 90% confidence interval [54, 55]. In addition, standardized residual correlations and modification indices were inspected to detect possible local dependence among the items.

Additionally, convergent validity was assessed as part of the construct validity of the ED-RRA. For this analysis, Pearson’s correlation coefficient and the Average Variance Extracted (AVE) value were examined. As a rule of thumb, the AVE value should be equal to or higher than 0.50 [56]. Following the logic of the Fornell-Larcker criterion, discriminant validity was appraised by comparing the Average Variance Extracted for the ED-RRA against the variance it shared (r²) with the RRS and the EDE-Q. Discriminant validity was additionally examined at the latent level. A two-factor model was specified in Sample 2, in which the ED-RRA was indicated by its 12 items and the EDE-Q by its four subscale scores, with the ED-RRA items again treated as ordinal indicators and the model estimated with WLSMV. Both factor variances were fixed at unity, so that the covariance between the factors could be read directly as their correlation, which was reported together with its 95% confidence interval. This model was then compared against a nested model in which the correlation was constrained to unity, that is, a model treating the two constructs as indistinguishable. Because WLSMV yields a mean- and variance-adjusted test statistic, the two models were compared through the DIFFTEST procedure rather than through an ordinary chi-square difference test.

To examine the incremental validity of the ED-RRA, a hierarchical multiple regression was conducted with the EDE-Q global score as the criterion. RRS, PHQ-9, and GAD-7 were entered at the first step, and the ED-RRA was added at the second step, so that the unique variance attributable to ED-specific rumination over and above general rumination and affective distress could be evaluated. Multicollinearity was assessed using tolerance and VIF values.

The reliability of the ED-RRA was assessed through both internal consistency and test-retest reliability. The Cronbach’s alpha coefficient is commonly used to test the internal consistency of a scale, and values greater than 0.70 are acceptable, while values greater than 0.80 indicate good reliability [57]. McDonald’s Omega coefficient is regarded as one of the most effective methods for determining reliability, with values of 0.80 or higher indicating good internal consistency [58]. To assess test-retest reliability, participants were voluntarily recruited from a university class. Sixty-two participants agreed to complete the ED-RRA again after 15 days, and 53 of them returned completed responses. To match responses across both time points while maintaining anonymity, participants created a self-generated nickname at each administration. No personal identifying information was collected or linked to study data. Test-retest reliability was quantified with an intraclass correlation coefficient (ICC) for single measurements, based on absolute agreement, and reported together with its 95% confidence interval. Absolute agreement was preferred over consistency, as it remains sensitive to systematic change across the two administrations. A paired-samples t-test was conducted alongside it to determine whether scores shifted systematically over the retest interval.

## Results

### Sample characteristics

In Sample 1, 392 participants (286 female) completed the 12-item ED-RRA. The mean age was 27.47 years (SD = 8.49; range = 18–65). In Sample 2, 387 participants (307 female) completed the ED-RRA and other measures. The mean age was 24.21 years (SD = 6.08; range = 18–65). Table 1 displays descriptive statistics for both samples.

To contextualize the level of ED symptomatology in Sample 2, EDE-Q global scores (mean format, range 0–6) were examined against established cut-offs. The mean global score was relatively low (M = 1.17, SD = 1.10), and the distribution was positively skewed (skewness = 1.18), with scores spanning the full range from 0 to 5.40. Only a small minority of participants (1.8%) reached the conventional clinical threshold of 4.0. By contrast, 16.5% scored at or above the community screening threshold of 2.3, derived in an Australian community sample of women [59]. This indicates that, although the sample was predominantly located in the lower-to-moderate symptom range, it nonetheless captured meaningful variability extending toward clinically relevant levels rather than being confined to a single extreme.

### Findings from validity analyses

#### Content validity

An analysis of the expert ratings showed that 12 items (item1, item2, item4, item6, item7, item8, item11, item12, item13, item14, item15, and item19) reached a CVR of 1.00, exceeding the critical threshold of ≥ 0.99 and indicating unanimous endorsement by all seven experts. The remaining items showed varying degrees of content validity, with CVR values ranging from − 0.14 to 0.71. One item (item 10) received a negative CVR value (-0.14), suggesting poor content validity. Most items (20 items) received a CVR value of 0.43, indicating moderate agreement among experts (see Supplementary Table S3 for details). The scale-level CVI for the final 12-item version was 1.00. The 12 retained items were exactly those that met the critical CVR threshold; qualitative expert evaluations, reported for each item in Supplementary Table S3, provided the conceptual rationale for the removal of each below-threshold item and informed the final wording of retained items where appropriate. Taken together with the high overall CVI, these results indicate that the final item set demonstrates strong content validity. This process ensured that the final items adequately represented the construct measured by the ED-RRA.

#### Construct validity

##### Item analysis

Pearson correlation analysis showed that the correlation coefficients between each of the 12 items and the total ED-RRA score were all above 0.30 (*p* < .001), suggesting that each item contributed meaningfully to the construct measured by the scale (see Table 2).

**Table 2:** Results of item analysis for the ED-RRA (Sample 1, *n* = 392)

Items	M	SD	Correlation coefficient between the item and the total score	Cronbach’s Alpha coefficient (α)	McDonald’s omega (ω)
**Mean item score**	2.05	0.88	1	0.94	0.94
**1.** Beden şeklim, kilom veya yeme davranışlarım ile ilgili olumsuz düşünceleri zihnimden bir türlü atamam.	2.39	1.14	0.77^**^		
**2.** “Keşke o kadar yemeseydim”, “keşke istediğim kiloda olsaydım” gibi düşünceler zihnimi işgal eder.	2.32	1.26	0.78^**^		
**4.** Beden şeklim, kilom veya yeme davranışlarım ile ilgili ufak değişimleri bile zihnimde takip ederim, yorumlarım (örn. aynaya baktığımda, giysi denediğimde).	2.63	1.20	0.73^**^		
**6.** Beden şeklim, kilom veya yeme davranışlarım ile ilgili sorunların sosyal hayatımı etkilediği düşüncesinden kurtulamam.	1.88	1.11	0.80^**^		
**7.** Günün büyük bir kısmını aldığım kalorileri nasıl yakacağımı düşünerek geçiririm (örn. egzersiz mi yapsam, bağırsak çalıştırıcı takviyeler mi kullansam gibi).	1.75	1.02	0.75^**^		
**8.** Günün büyük bir kısmını öğünlerde nasıl besleneceğimi düşünerek geçiririm (örn. ne kadar yiyeceğim, ne zaman yiyeceğim, kaç öğün yiyeceğim?).	2.12	1.15	0.69^**^		
**11.** Beden şeklim, kilom veya yeme davranışlarım ile ilgili sorunların romantik ilişkilerimi etkilediği düşüncesinden kurtulamam.	1.86	1.11	0.70^**^		
**12.** İdeal kiloda ve beden şeklinde olduğum, yeme davranışımı kontrol edebildiğim senaryolar hayal ederim.	2.17	1.20	0.84^**^		
**13.** Beden şeklimi, kilomu veya yeme davranışlarımı kontrol etmeye çalışırken kendimi hoşlandığım yiyecekleri düşünmekten alıkoyamam.	2.06	1.18	0.77^**^		
**14.** Düşünmemeye çalışsam da beden şeklim, kilom veya yeme davranışlarım ile ilgili başarısızlıklarım aklıma gelir.	1.88	1.10	0.84^**^		
**15.** Günün büyük bir kısmında beden şeklim, kilom veya yeme davranışlarım ile ilgili yetersizliklerimi düşünüp dururum.	1.75	1.00	0.85^**^		
**19.** Beden şeklim, kilom veya yeme davranışlarım ile ilgili kontrolü kaybedeceğim düşüncesini bir türlü zihnimden atamam.	1.75	1.07	0.83^**^		

Notes: **p < .001; For full item content in Turkish and English, see Supplementary Material Table S1

To examine the possibility of item redundancy, the inter-item correlation matrix was also inspected. The mean inter-item correlation was *r* = .57 (range = 0.40–0.79), indicating that the items were strongly homogeneous in content, as would be expected for a narrowly defined unidimensional construct. The highest correlation, observed between Items 14 and 15 (*r* = .79), nonetheless remained below the threshold conventionally taken to indicate problematic redundancy, and no item pair exceeded *r* = .80.

### Exploratory factor analysis (EFA)

Prior to the factor analysis, the distributional properties of the items were examined. Item-level skewness ranged from 0.34 to 1.33, and floor responses were common, as the lowest category (“Never”) was selected by between 19.1% and 58.7% of participants across the individual items. Consistent with this pattern, Mardia’s tests indicated significant departures from multivariate normality. Multivariate skewness was b₁ₚ = 22.06, with an associated χ²(364) = 1441.50, *p* < .001. Multivariate kurtosis was b₂ₚ = 223.92, against an expected value of 168 under multivariate normality, which yields z = 30.20, *p* < .001. These results supported the treatment of the items as ordinal indicators and the use of WLSMV estimation based on a polychoric correlation matrix.

As a preliminary check on the factorability of the data, the KMO measure was 0.94 and Bartlett’s test of sphericity was significant (χ² = 3270.489, *p* < .001), indicating that the data were suitable for factor analysis. The analysis of the polychoric correlation matrix then yielded a single eigenvalue greater than 1.00 (8.142), whereas all subsequent eigenvalues were well below this threshold, with the second eigenvalue being 0.74. The first factor accounted for 67.85% of the variance. Standardized factor loadings were uniformly high, ranging from 0.71 to 0.92, as shown in Table 3. The observed eigenvalues showed a sharp decline after the first factor, consistent with the scree criterion.

**Table 3:** Results of exploratory factor analysis for the ED-RRA (Sample 1, *n* = 392)

Items	Factor	Eigenvalue	Explained variance (%)
Total Factor		8.142	67.85
Items 1	0.78		
Items 2	0.79		
Items 4	0.73		
Items 6	0.83		
Items 7	0.80		
Items 8	0.71		
Items 11	0.75		
Items 12	0.86		
Items 13	0.80		
Items 14	0.90		
Items 15	0.92		
Items 19	0.88		

Notes: Extraction was based on a polychoric correlation matrix using the WLSMV estimator. No rotation was applied to the single-factor solution

**Fig. 2:**
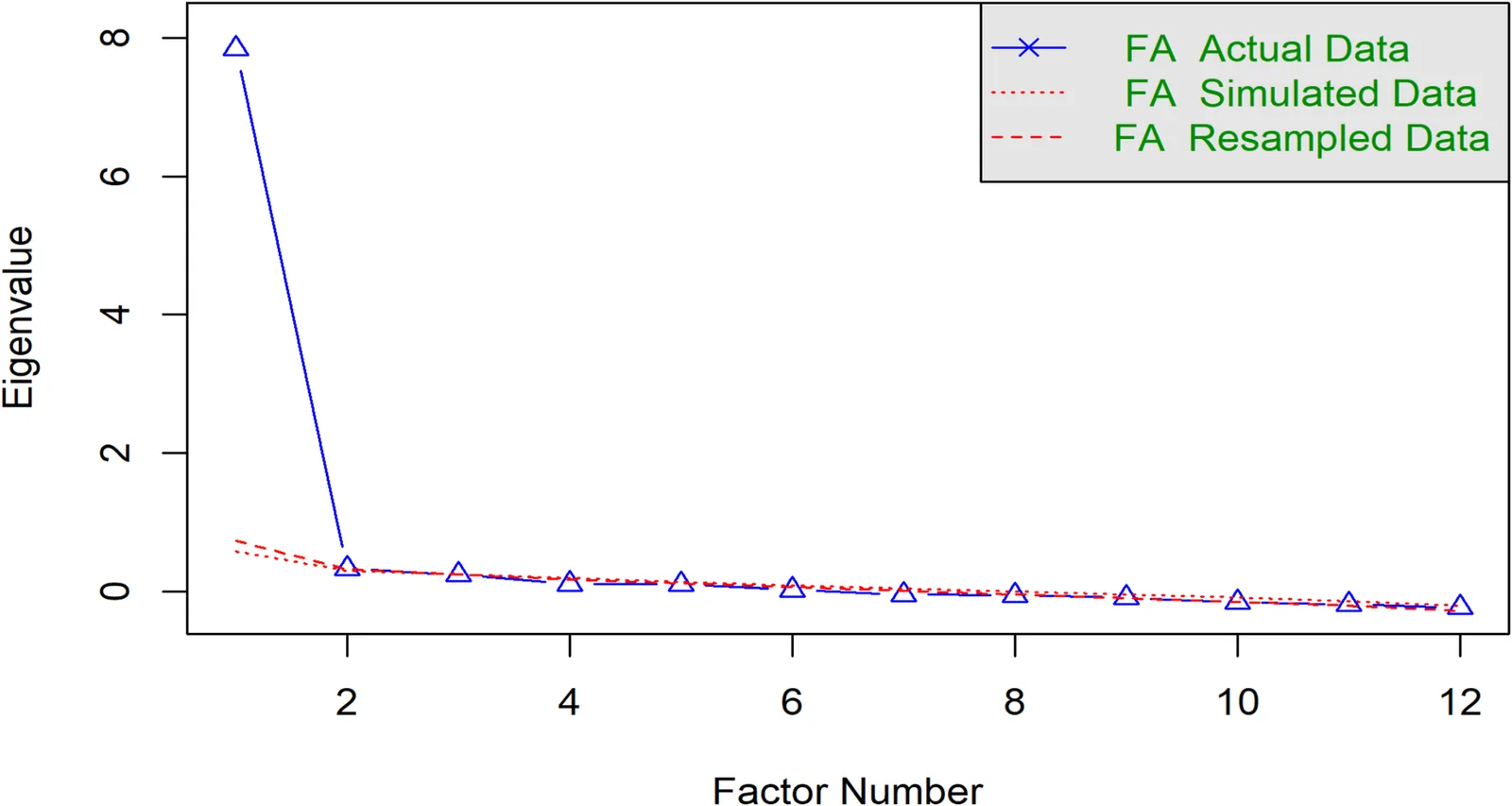
Parallel analysis based on the polychoric correlation matrix for the ED-RRA (Sample 1, *n* = 392). The observed eigenvalues (actual data) are plotted against those obtained from simulated and resampled random data. Only the first eigenvalue exceeded its random counterpart, supporting a single-factor solution

Parallel analysis based on the polychoric correlation matrix converged with these results (Fig. 2). Only the first eigenvalue obtained from the sample exceeded the 95th percentile of the eigenvalues generated from 500 random data sets, whereas the second and all subsequent eigenvalues fell at or below the corresponding random values. Taken together, the eigenvalue-greater-than-one criterion, the scree plot, as well as the parallel analysis consistently supported a unidimensional structure for the ED-RRA.

### Confirmatory factor analysis (CFA)

**Fig. 3:**
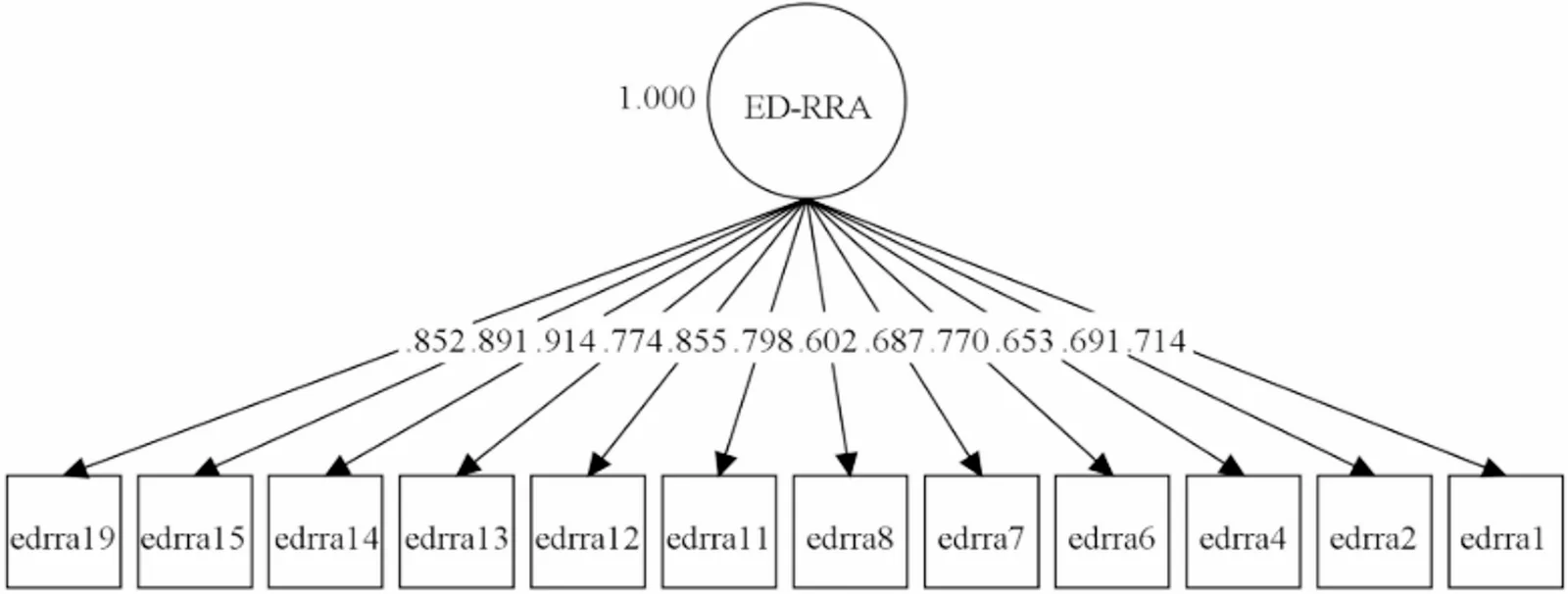
Standard one factor structural model for the ED-RRA (Sample 2, *n* = 387)

The single-factor structure identified in Sample 1 was tested on Sample 2 (*n* = 387) using CFA with WLSMV estimation. The model showed generally adequate fit, with a scaled χ²(54) = 202.283, *p* < .001, CFI = 0.980, TLI = 0.975, SRMR = 0.034, alongside a marginal RMSEA of 0.084 (90% CI [0.072, 0.097]) that exceeded the prespecified criterion of 0.08. Standardized factor loadings were all substantial and statistically significant, ranging from 0.60 to 0.91 (*p* < .001), as shown in Table 4; Fig. 3. Inspection of the standardized residual correlations revealed no substantial local dependence among the items. The largest residual correlation was small (0.16), and no modification index exceeded the threshold of 3.84. In particular, the residual correlation between Items 14 and 15, which had shown the highest inter-item correlation, was negligible under the fitted model. Accordingly, the unidimensional model reproduced the item associations well, without any need for correlated residuals.

**Table 4:** Goodness-of-fit indices for the ED-RRA measurement models (Sample 2, *n* = 387)

Fit indices	Scaled χ² (df)	CFI	TLI	RMSEA [90% CI]	SRMR
Prespecified criterion	n/a	≥ 0.90	≥ 0.90	≤ 0.08	≤ 0.08
Single-factor ED-RRA model	202.283 (54)*	0.980	0.975	0.084 [0.072, 0.097]	0.034
Two-factor model (ED-RRA and EDE-Q)	390.150 (103)*	0.951	0.943	0.085 [0.076, 0.094]	0.041
Two-factor model with the factor correlation fixed at unity	561.086 (104)*	0.922	0.910	0.107 [0.098, 0.115]	0.061

Notes:*p < .001. All models were estimated with the WLSMV estimator using a polychoric correlation matrix, with the ED-RRA items treated as ordinal indicators. Standardized factor loadings ranged from .60 to .91 in the single-factor model. In the two-factor models, the ED-RRA was indicated by its 12 items and the EDE-Q by its four subscale scores. The constrained model, in which the two factors were treated as indistinguishable, fitted significantly worse than the unconstrained model, Δχ²(1) = 125.83, p < .001 (DIFFTEST). The RMSEA values for the single-factor model as well as for the two-factor model exceeded the prespecified criterion of 0.08, and the fit of both models is accordingly described as generally adequate with a marginal RMSEA. Abbreviations. CFI = comparative fit index, EDE-Q = Eating Disorder Examination Questionnaire, ED-RRA = Eating Disorder-Related Rumination Assessment Scale, RMSEA = root mean square error of approximation, SRMR = standardized root mean square residual, TLI = Tucker-Lewis index

#### Convergent validity

Pearson correlation analysis for Sample 2 (*n* = 387) showed that the scores of ED-RRA were positively correlated with the scores of EDE-Q (*r* = .76; *p* < .001), PHQ-9 (*r* = .36; *p* < .001), GAD-7 (*r* = .37; *p* < .001), and RRS (*r* = .34; *p* < .001) (see Table 5). Beyond this global association with ED pathology, the convergent pattern was examined at the subscale level to determine whether the ED-RRA related disproportionately to particular domains of ED cognition (Table S2). The ED-RRA correlated most strongly with Shape Concern (*r* = .75) and Weight Concern (*r* = .72), with somewhat weaker associations for Eating Concern (*r* = .67) and Restraint (*r* = .54), all *p* < .001. Additionally, the analysis revealed an adequate Average Variance Extracted (AVE) of 0.60, which exceeds the conventional threshold of 0.50 and provides evidence for the convergent validity of the ED-RRA.

**Table 5:** Correlations between ED-RRA and other measures (Sample 2, *n* = 387)

	ED-RRA	EDE-Q	PHQ-9	GAD-7	RRS
ED-RRA	-				
EDE-Q	0.76^**^	-			
PHQ-9	0.36^**^	0.33^**^	-		
GAD-7	0.37^**^	0.37^**^	0.82^**^	-	
RRS	0.34^**^	0.32^**^	0.67^**^	0.74^**^	-

Notes: **p< 0.001. Abbreviations. EDE-Q = Eating Disorders Examination Questionnaire, GAD-7 = Generalized Anxiety Disorder Questionnaire, PHQ-9 = Patient Health Questionnaire, ED-RRA = Eating Disorder-Related Rumination Assessment Scale, RRS = Ruminative Responses Scale

#### Discriminant validity

The AVE of the ED-RRA (0.60) exceeded the variance shared with general depressive rumination (RRS, r² = 0.12) by a wide margin. By contrast, it exceeded the variance shared with ED pathology (EDE-Q, r² = 0.58) only marginally. In other words, the ED-RRA was clearly discriminable from general rumination, whereas the evidence for its discrimination from ED symptom severity remained borderline by this criterion.

The latent-variable analysis provided a more direct test of the distinction between the ED-RRA and ED symptom severity. The two-factor model showed generally adequate fit, with χ²(103) = 390.15, *p* < .001, CFI = 0.951, TLI = 0.943, SRMR = 0.041, as well as a marginal RMSEA of 0.085 (90% CI [0.076, 0.094]) (see Table 4). The correlation between the two factors was 0.77 (SE = 0.025, 95% CI [0.72, 0.82]), and the upper bound of this interval fell below the conventional criterion of 0.85 for distinguishable constructs. Constraining the correlation to unity produced a significant deterioration in fit, Δχ² (1) = 125.83, *p* < .001, alongside poorer values across the fit indices (CFI = 0.922, TLI = 0.910, RMSEA = 0.107, SRMR = 0.061). The two constructs are therefore not empirically interchangeable. The squared latent correlation (0.59) nonetheless remained close to the AVE, which indicates them to be closely related rather than clearly separable.

#### Incremental validity

A hierarchical regression was conducted to test whether the ED-RRA predicted ED pathology beyond general rumination and affective distress (see Table 6). At Step 1, general rumination, depression, and anxiety together accounted for a significant but modest proportion of the variance in EDE-Q global scores, R² = 0.13, F(3, 383) = 19.92, *p* < .001. The addition of the ED-RRA at Step 2 produced a substantial and significant increment, ΔR² = 0.44, ΔF(1, 382) = 401.56, *p* < .001, raising the total explained variance to R² = 0.58. In the final model, the ED-RRA was the only significant predictor (β = 0.72, *p* < .001), whereas the contributions of general rumination (β = 0.00, *p* = .98), depression (β = −0.01, *p* = .88), and anxiety (β = 0.09, *p* = .16) were no longer significant. All VIF values were below 4.0 (range 1.18–3.95), indicating that multicollinearity did not threaten the stability of the estimates.

**Table 6:** Hierarchical regression predicting EDE-Q global scores from the ED-RRA over general rumination and affective distress (Sample 2, *n* = 387)

Predictor	β (Step 1)	β (Step 2)	VIF (Step 2)
RRS	0.10	0.00	2.31
PHQ-9	0.09	−0.01	3.18
GAD-7	0.21*	0.09	3.95
ED-RRA	—	0.72***	1.18
R²	0.13	0.58	
ΔR²	0.13***	0.44***	
ΔF	19.92***	401.56***	

Note: Standardized regression coefficients (β) are reported. VIF = variance inflation factor. ΔF refers to the incremental F test associated with the change in R². Degrees of freedom are (3, 383) at Step 1 and (4, 382) at Step 2 for the model tests, as well as (1, 382) for ΔF at Step 2.*p < .05, ***p < .001

### Findings from reliability analysis

In Sample 2, Cronbach’s alpha was 0.93 and McDonald’s omega was 0.93 for the single-factor structure of the ED-RRA. Among the 53 participants who completed the retest, the ICC for single measurements, based on absolute agreement, was 0.88 (95% CI [0.77, 0.94]), indicating good temporal stability. Scores nonetheless declined across the two administrations, from M = 25.60 (SD = 9.52) to M = 23.53 (SD = 10.05). This difference of 2.08 points reached significance in a paired samples t-test, t (52)= 3.48, *p* = .001 (95% CI [0.88, 3.27]).

## Discussion

This study aimed to develop a measure to assess the severity of rumination specific to ED symptomatology and to evaluate its psychometric properties in a Turkish non-clinical adult sample. There is no scale adapted or developed in Türkiye to assess rumination specific to eating disorders. Although a measure of ED-specific rumination (RRS-ED; [15]) already exists and could be adapted into Turkish, this scale was developed by only minimally modifying a general depressive rumination assessment. Accordingly, rather than relying on general depressive rumination constructs, we aimed to assess dimensions of rumination that more directly reflect the motivational and cognitive dynamics described in Goal Progress Theory [19]. It is in this conceptual reorientation, rather than in the measurement of an altogether new construct, that the novelty of the ED-RRA resides. A conceptual inspection of the RRS-ED items supports this distinction at the item level. They largely represent passive, affect-focused dwelling on eating-related distress, consistent with their origin as a minimally modified adaptation of the general depressive-rumination framework [18], rather than a reconceptualization oriented toward goal-related processes. By contrast, the ED-RRA items were developed to capture goal-related and control-focused ruminative processes, including preoccupation with unmet standards, perceived loss of control, and cyclical self-monitoring. These processes follow from the goal-based account of rumination on which the ED-RRA was built [19], although they are not structurally represented in the RRS-ED.

This conceptual distinction, however, does not extend the scope of the present evidence. The results are best read as establishing the preliminary psychometric adequacy of the ED-RRA within a non-clinical context. They do not, by themselves, license the use of the scale for clinical screening, diagnostic classification, or therapeutic decision-making, each of which awaits validation in formally diagnosed samples.

The Goal Progress Theory emphasizes that rumination intensifies when individuals experience failure in progressing toward personally salient, higher-order goals. In line with this, we focused on the following key content areas commonly reflected in ruminative thinking in eating disorders: self-oriented and socially prescribed perfectionism, rigid goals, intense control efforts, and comparisons related to higher-order goals; tendencies to imagine alternative scenarios involving these goals; experiences of perceived failure and inadequacy in relation to higher-order goals; and the cognitive costs associated with the deliberate suppression of certain behaviours or thoughts.

The scale development process followed the framework proposed by DeVellis and Thorpe [30]. The 12-item ED-RRA demonstrated adequate reliability and validity, with factor analyses supporting a unidimensional structure. During the development of the ED-RRA, the theoretical construct of rumination and its relevance in the context of eating disorders were systematically evaluated and expert input from clinicians working in the field was utilized to ensure that the scale would be both conceptually relevant and sensitive within the target cultural context. Expert evaluations further supported the content relevance of the items [32]. Nevertheless, this finding may reflect both item content tailored to ED-specific cognitive patterns and characteristics of the Turkish community sample. As such, further validation studies of the ED-RRA across different cultural populations are warranted to evaluate the replicability of the factor structure and to better delineate whether the one-dimensionality of the ED-RRA is derived from sample characteristics or from the broad and specific nature of its items.

The factor-analytic findings warrant a measured interpretation. Because the items are ordinal and their distributions departed from multivariate normality, the analyses were estimated from a polychoric correlation matrix using a robust categorical estimator rather than maximum likelihood. Under this approach, the exploratory analysis, the scree plot, as well as a parallel analysis converged on a single factor, and the single-factor structure was subsequently tested on the second sample. The RMSEA for the confirmatory model exceeded the prespecified criterion of 0.08. Interpreted alongside the strong CFI, TLI, and SRMR values, as well as the absence of substantial residual correlations or modification indices, the single-factor model is best described as showing generally adequate fit with a marginal RMSEA rather than excellent fit. Its structure therefore warrants replication in independent, and ideally clinical, samples. The pronounced floor responding observed for several items warrants comment. In a non-clinical sample, a concentration of responses in the lowest category is to be expected for items indexing ED-specific ruminative content, rather than reflecting a deficiency in item quality. In other words, the floor pattern is consistent with a community sample situated at the lower end of the ruminative continuum. Accordingly, this distributional feature does not undermine the psychometric performance of the scale, although it does reinforce the need for replication in samples with greater representation of clinically significant ED psychopathology, in which a broader range of responding would be anticipated.

Relatedly, the strong internal consistency and clear single-factor solution should themselves be interpreted with some caution. Because the item pool was reduced through a stringent, consensus-driven content-validity procedure (a required CVR of ≥ 0.99 for a seven-member panel, with an overall CVI of 1.00), the retained items are necessarily homogeneous in content. Consistent with this, the mean inter-item correlation (*r* = .57) fell somewhat above the range commonly recommended for a homogeneous yet non-redundant item set (0.15–0.50; [60]). This pattern suggests that the observed unidimensionality and the high reliability estimates partly reflect content overlap among items rather than construct breadth alone. Consequently, some less prototypical, yet theoretically meaningful, aspects of ED-specific rumination may be underrepresented in the final item set. Nevertheless, most removed items are considered to have reflected overlapping content or conceptually distinct processes rather than additional facets of ED-specific rumination. Although no single item pair reached a level indicative of outright redundancy, the ED-RRA is best understood as a coherent measure of a deliberately narrow construct. The extent to which it captures the full breadth of ED-specific ruminative content (as opposed to a highly cohesive core) thus warrants further examination in future work.

In addition, the findings indicate that the ED-RRA demonstrated excellent internal consistency, with all items showing strong item–total correlations and standardized factor loadings ranging from 0.60 to 0.91. Further psychometric analyses supported the convergent validity of the ED-RRA, as evidenced by significant and positive correlations with depression (PHQ-9), anxiety (GAD-7), general rumination (RRS), and eating disorder symptoms (EDE-Q). These findings support previous research showing a relationship between rumination and increased eating disorders and general psychopathology [4, 6]. Moreover, the strongest relationship was reported with the EDE-Q (*r* = .76). In EDs, high-level goals are typically related to ideal weight, body shape, and eating behaviour; therefore, repetitive thought content focused on control efforts forms a central component of the rumination cycle. Although the EDE-Q is similar in content as it assesses the severity of ED symptoms, the ED-RRA directly measures the intrusive, repetitive, and persistent nature of rumination. The differential pattern of subscale correlations is consistent with this theoretical emphasis. The construct was framed around higher-order goals concerning ideal weight and body shape, as well as the control efforts these entail. Accordingly, it correlated most strongly with the shape- and weight-concern domains of the EDE-Q rather than with dietary restraint. In other words, the ED-RRA appears to capture goal- and evaluation-focused ruminative content rather than the behavioural regulation of eating per se. Similarly, a principal component analysis with varimax rotation, in which RRS-ED and EDE-Q items were entered together, yielded five components. The RRS-ED brooding and reflection items formed two components of their own, whereas the EDE-Q items formed three further components, labelled shape/weight concern, restraint, and eating. The eating component, however, also included a small number of restraint and shape items. Together, these five components accounted for 67.2% of the variance [15]. In other words, ED-specific rumination and ED symptomatology emerged as related but separable constructs, each cohering within its own conceptual domain. This finding reveals that both RRS-ED and EDE-Q items are consistent and highly correlated within their conceptual domains. Additionally, small-to-moderate correlations were observed with measures of depression, anxiety, and general rumination (r = from 0.34 to 0.37). These correlations align with theoretical expectations, share some conceptual overlap, yet remain distinct from broader affective and cognitive constructs [6].

Taken together, this pattern of associations warrants an explicit statement of our conceptual position, since it can be read in more than one way. We conceptualize ED-specific rumination not as a content domain separate from eating pathology, but as a domain-specific repetitive thinking process. On this view, the characteristic features of rumination (particularly its uncontrollable quality and its fixation on perceived goal discrepancies) are applied to the content domain of eating, weight, and shape. From this standpoint, the observed correlations are not merely compatible with content specificity; they are precisely what such an account predicts. Because the ED-RRA and the EDE-Q are anchored in the same content domain, a strong association between them is expected. The comparatively modest correlation with the RRS, by contrast, follows from the fact that general rumination, although sharing the process features of repetitive thought, is anchored in depressive rather than eating-related content. The strong EDE-Q correlation and the modest RRS correlation thus form the expected signature of a domain-specific ruminative process rather than an artefact of content dependency. At the same time, the magnitude of the EDE-Q association raises a legitimate question of discriminant validity, which we address below.

The hierarchical regression is informative in two respects. On the one hand, the ED-RRA predicted ED pathology well beyond the variance explained by general rumination, depression, and anxiety, accounting for a substantial unique increment and emerging as the sole significant predictor once these constructs were held constant. This is consistent with our contention that the ED-RRA indexes ruminative content specific to eating, weight, and shape. That is, it reflects neither generic repetitive thought nor affective distress. On the other hand, the magnitude of this increment partly reflects the considerable shared variance between the ED-RRA and the EDE-Q, and the analysis should not be read as evidence that ED-specific rumination is fully separable from ED cognitive preoccupation. Rather, the two instruments occupy adjacent conceptual territory: the ED-RRA captures the ruminative process directed at eating-related goals, whereas the EDE-Q indexes the broader severity of eating pathology.

The latent-variable analysis bears more directly on the separability of the two constructs than the Fornell-Larcker comparison does. Once measurement error was taken into account, the two constructs correlated at 0.77, and a model treating them as one deteriorated significantly in fit. In other words, ED-specific rumination and ED symptom severity are not interchangeable at the latent level. The squared latent correlation nonetheless remained close to the AVE for the ED-RRA. Accordingly, the two are best described as empirically distinguishable yet closely related, rather than as clearly separable domains. The increment observed in the hierarchical regression should be read in the same light. Its magnitude reflects a genuine predictive contribution as well as substantial content overlap, and it does not on its own establish the ED-RRA to index a process independent of ED cognitive severity.

This distinction is visible at the item level. The ED-RRA items assess the recurrence of thought despite deliberate suppression (Item 14), its intrusive quality (Items 1 and 6), as well as its temporal dominance across the day (Item 15). By contrast, the majority of EDE-Q items assess the frequency of discrete behaviours over the preceding 28 days, or else a single evaluative judgment at one point in time. In other words, the two instruments differ in what they ask respondents to report, although they are anchored in the same content domain. What the present increment cannot establish, given a single-occasion self-report design, is the separability of ED-specific rumination from ED cognitive severity against a less content-overlapping criterion. Establishing incremental validity against clinician ratings, behavioural indices, or longitudinal and treatment-response outcomes therefore remains an essential task for future research. In particular, future studies could examine whether ED-RRA scores predict treatment non-response or symptom persistence over and above baseline EDE-Q severity, which would provide evidence of incremental validity that is free of criterion overlap.

Reliability findings indicate the ED-RRA to offer high internal consistency as well as good temporal stability. Cronbach’s α and McDonald’s ω demonstrate the scale’s strong internal reliability, while the ICC indicates scores to remain reasonably stable across a short-to-medium interval. A small yet significant decline in mean scores was nevertheless observed between the two administrations. Such a decline is consistent with repeated administration effects documented for self-report symptom measures, although the present design cannot separate these from regression to the mean. Whether ED-specific rumination behaves as a durable trait over longer periods awaits assessment with extended retest intervals.

### Limitations

This study has several limitations that should be noted. The sample characteristics may restrict the generalizability of the findings. The study was conducted with a non-clinical sample consisting primarily of females within a normal BMI range, which may not fully capture the characteristics of individuals with clinical EDs. Consistent with this, the distribution of EDE-Q scores in the present sample indicated predominantly low-to-moderate levels of ED symptomatology. Only a small proportion reached conventional clinical thresholds. Although a non-negligible minority reported symptom levels above community-based cut-offs, the relatively low mean and positive skew suggest the psychometric performance of the ED-RRA to have been established largely within a non-clinical, lower-symptom range. The same constraint is visible in the floor responding discussed above. ED-specific rumination may nonetheless differ in its content, severity, and functional significance among individuals with clinically diagnosed eating disorders, so the psychometric properties observed here may not fully generalize to clinical ED populations. Studies directly comparing clinical and non-clinical samples are therefore a priority, so that the extent to which the factor structure, score distribution, and item functioning of the ED-RRA generalize across diagnostic status can be established rather than assumed.

However, previous research suggests that a considerable proportion of individuals with clinically significant ED psychopathology do not meet formal diagnostic criteria for a specific ED, and those with subthreshold EDs often display levels of severity comparable to individuals with full-threshold diagnoses [61]. Moreover, key cognitive and behavioural features of ED psychopathology, including body dissatisfaction and binge eating, have been widely documented in non-clinical and community populations [34–36]. Relatedly, the present cross-sectional design cannot speak to the scale’s predictive validity, its sensitivity to change, or its clinical utility. These properties are central to applied use, yet establishing them calls for longitudinal and treatment-based designs rather than a single-occasion validation. The practical significance of the ED-RRA should therefore be regarded as provisional until such evidence accumulates.

The samples were also markedly imbalanced with respect to gender, with women comprising the clear majority in both the EFA and CFA subsamples. Given the relatively small number of male participants, a formal test of measurement invariance across gender could not be conducted with adequate statistical power in the present data, and the cross-gender stability of the single-factor structure therefore remains an open question. Establishing configural, metric, and scalar invariance across gender and, ideally, across age groups, represents an important direction for subsequent research with more balanced samples, particularly in light of the gender-related distribution of eating-disorder psychopathology.

A further constraint concerns the recruitment strategy. Participants were reached through voluntary online snowball sampling, an approach that, while practical for accessing a sizeable community sample during an initial validation, does not yield a probabilistically representative sample and may introduce selection bias, since participants who elect to take part in studies concerning eating and body-related concerns may differ systematically from those who do not. The demographic and psychological profile of the present sample should therefore not be read as representative of the wider Turkish adult population, and replication using probability-based or stratified sampling would strengthen confidence in the generalizability of the ED-RRA’s psychometric properties.

In addition, the pilot phase was limited to clarity and acceptability testing and did not include a formal cognitive-interviewing protocol designed to examine participants’ cognitive response processes. Future studies should therefore employ cognitive interviewing, including think-aloud procedures and systematic verbal probing, to examine how respondents interpret the ED-RRA items and formulate their responses, and to identify any potential sources of response-process error.

All constructs in the present study were assessed through self-report. This raises the possibility that the observed associations were inflated to some degree by shared method variance, as well as by response biases such as social desirability. In addition, no dedicated attention-check or validity items were embedded in the battery, so careless or inattentive responding could not be screened directly. Future work would benefit from incorporating attention-check items and from triangulating self-report data with behavioural indices, as well as with clinician-rated assessments of ED-specific rumination. Relatedly, temporal stability was evaluated over a 15-day interval. This interval, although adequate for establishing short-term reliability, does not speak to the longer-term stability that would be expected of a trait-like construct. In other words, the present design captures consistency across days, yet leaves the stability across months or years an open question. Future studies employing longer retest intervals are thus needed to characterize the temporal dynamics of ED-specific rumination more fully.

Although the ED-RRA demonstrated satisfactory psychometric properties in a Turkish adult sample, its applicability to other linguistic and cultural contexts remains to be tested. Future research should examine the validity and factor structure of the ED-RRA in clinical populations and across diverse cultural groups to ensure its broader utility and cross-cultural relevance [35, 36].

The RRS-ED was not included in the assessment battery, and no validated Turkish adaptation of it is currently available. A direct head-to-head comparison, including incremental and discriminant validity against the most relevant competing ED-specific measure, could therefore not be conducted. Adapting the RRS-ED was not considered a viable alternative in this respect. The RRS-ED is grounded in a depressive rumination framework, which may not adequately reflect the goal-related cognitive processes, control efforts, and perfectionistic concerns central to rumination in eating [18, 19, 62]. By contrast, the ED-RRA was developed on the basis of Goal Progress Theory, aiming to assess these more specific and theoretically grounded aspects of ED-related rumination. Indirect support for the content specificity anticipated by the goal-progress account nonetheless emerged from the pattern of associations, as the ED-RRA related strongly to ED pathology although its association with general rumination remained modest. The added value of the ED-RRA over the RRS-ED nevertheless remains to be established empirically. Accordingly, the present contribution should be understood as the introduction of a theoretically distinct, standalone measure rather than as a demonstration of its superiority, and a direct comparison is a priority for future work once a validated Turkish RRS-ED becomes available.

## Conclusions

In conclusion, the present findings indicate the ED-RRA to show promising preliminary psychometric properties as a measure of ruminative processes specific to EDs. The scale demonstrated excellent internal consistency, a coherent unidimensional factor structure, and theoretically consistent associations with related psychological constructs. These findings indicate that the ED-RRA may serve as a useful instrument for identifying ED-related rumination in non-clinical populations. However, additional validation in clinical samples and across culturally diverse groups is necessary to determine its generalizability and to clarify its potential role in diagnostic and therapeutic contexts.

## Supplementary information

Below is the link to the electronic supplementary material.

Supplementary Material 1

## Data Availability

To protect personal data, the datasets utilized in this study are not publicly available. To access these datasets, please contact the author directly at [caner.dogrusever@siirt.edu.tr](mailto: caner.dogrusever@siirt.edu.tr).

## Declarations

### Public significance statement

This study developed the Eating Disorder-Related Rumination Assessment Scale (ED-RRA) and examined its initial psychometric properties in a Turkish non-clinical adult sample. The scale was designed to assess goal-related and control-focused ruminative processes in the context of eating, weight, and body shape. It can support future research on the cognitive processes linked to ED symptoms. Validation in formally diagnosed samples is required before the scale can be considered for clinical use.
