# Supplementary material for: Development and initial validation of the eating disorder-related rumination assessment scale in a Turkish non-clinical sample
Source: BMC Psychol. 2026 Aug 26;14:1549. doi: 10.1186/s40359-026-05457-7 (PMC13711953; doi:10.1186/s40359-026-05457-7)
Supplement: Supplementary file 1 [file 40359_2026_5457_MOESM1_ESM.docx]

**Supplementary Materials**

**Table S1.** Eating Disorder-Related Rumination Assessment Scale (ED-RRA): Turkish Version and English Translation

| **Instructions (Turkish):**  Aşağıdaki ifadeler “BEDEN ŞEKLİNİZ, KİLONUZ veya YEME DAVRANIŞLARINIZ” ile ilgilidir. Lütfen “SON BİR AY İÇİNDE” bu ifadeleri ne sıklıkla düşündüğünüzü, yaptığınızı değerlendirin ve “1= Hiçbir Zaman, 2=Nadiren, 3= Ara Sıra, 4=Çoğunlukla, 5=Her Zaman” seçenekleri arasından size en uygun olan kısmı işaretleyiniz.  **Instructions (English):**  The following statements relate to YOUR BODY SHAPE, WEIGHT, or EATING BEHAVIOURS. Please consider how often you have thought about or done these things IN THE LAST MONTH and select the most appropriate option: "1 = Never, 2 = Rarely, 3 = Sometimes, 4 = Often, 5 = Always."  **Response Scale:**  1 = Never / Hiçbir Zaman, 2 = Rarely / Nadiren, 3 = Sometimes / Ara Sıra, 4 = Often / Çoğunlukla, 5 = Always / Her Zaman | | | | | | |
| --- | --- | --- | --- | --- | --- | --- |
| Item 1 | **Turkish:** Beden şeklim, kilom veya yeme davranışlarım ile ilgili olumsuz düşünceleri zihnimden bir türlü atamam.  **English:** I cannot get negative thoughts about my body, weight or eating behaviours out of my mind. | **1** | **2** | **3** | **4** | **5** |
| Item 2 | **Turkish:** “Keşke o kadar yemeseydim”, “keşke istediğim kiloda olsaydım” gibi düşünceler zihnimi işgal eder.  **English:** Thoughts like “I wish I hadn’t eaten so much” and “I wish I was at the weight I want to be” occupy my mind. | **1** | **2** | **3** | **4** | **5** |
| Item 4 | **Turkish:** Beden şeklim, kilom veya yeme davranışlarım ile ilgili ufak değişimleri bile zihnimde takip ederim, yorumlarım (örn. aynaya baktığımda, giysi denediğimde).  **English:** I monitor and analyse even the smallest changes in my body shape, weight or eating behaviours (e.g., when I look in the mirror or try on clothes) | **1** | **2** | **3** | **4** | **5** |
| Item 6 | **Turkish:** Beden şeklim, kilom veya yeme davranışlarım ile ilgili sorunların sosyal hayatımı etkilediği düşüncesinden kurtulamam.  **English:** I cannot get rid of the idea that problems with my body shape, weight or eating behaviours affect my social life. | **1** | **2** | **3** | **4** | **5** |
| Item 7 | **Turkish:** Günün büyük bir kısmını aldığım kalorileri nasıl yakacağımı düşünerek geçiririm (örn. egzersiz mi yapsam, bağırsak çalıştırıcı takviyeler mi kullansam gibi).  **English:** I spend most of the day thinking about how to burn off all the calories I ate (e.g., should I exercise or use laxatives). | **1** | **2** | **3** | **4** | **5** |
| Item 8 | **Turkish:** Günün büyük bir kısmını öğünlerde nasıl besleneceğimi düşünerek geçiririm (örn. ne kadar yiyeceğim, ne zaman yiyeceğim, kaç öğün yiyeceğim?).  **English:** I spend most of the day thinking about my meals (e.g., what to eat, how much to eat, when to eat, and how many meals I should have?). | **1** | **2** | **3** | **4** | **5** |
| Item 11 | **Turkish:** Beden şeklim, kilom veya yeme davranışlarım ile ilgili sorunların romantik ilişkilerimi etkilediği düşüncesinden kurtulamam.  **English:** I cannot get rid of the idea that problems with my body shape, weight or eating behaviours affect my romantic relationships. | **1** | **2** | **3** | **4** | **5** |
| Item 12 | **Turkish:** İdeal kiloda ve beden şeklinde olduğum, yeme davranışımı kontrol edebildiğim senaryolar hayal ederim.  **English:** I imagine scenarios where I am of ideal weight and body shape and have control over my eating behaviour. | **1** | **2** | **3** | **4** | **5** |
| Item 13 | **Turkish:** Beden şeklimi, kilomu veya yeme davranışlarımı kontrol etmeye çalışırken kendimi hoşlandığım yiyecekleri düşünmekten alıkoyamam.  **English:** When trying to control my body shape, weight or eating behaviours, I cannot stop myself from thinking about the foods I enjoy. | **1** | **2** | **3** | **4** | **5** |
| Item 14 | **Turkish:** Düşünmemeye çalışsam da beden şeklim, kilom veya yeme davranışlarım ile ilgili başarısızlıklarım aklıma gelir.  **English:** Despite trying not to think about it, thoughts of failing to achieve my body shape, weight, or eating goals keep coming back to my mind. | **1** | **2** | **3** | **4** | **5** |
| Item 15 | **Turkish:** Günün büyük bir kısmında beden şeklim, kilom veya yeme davranışlarım ile ilgili yetersizliklerimi düşünüp dururum.  **English:** I spend most of the day thinking that I am not good enough because of my body shape, weight, or eating behaviours. | **1** | **2** | **3** | **4** | **5** |
| Item 19 | **Turkish:** Beden şeklim, kilom veya yeme davranışlarım ile ilgili kontrolü kaybedeceğim düşüncesini bir türlü zihnimden atamam.  **English:** I cannot get rid of the thought that I will lose control over my body shape, weight or eating behaviours. | **1** | **2** | **3** | **4** | **5** |

**Note.** The ED-RRA was developed and validated in Turkish. English translations are provided for interpretive purposes only and should not be used for assessment without formal validation. The English versions have not undergone psychometric evaluation and are not intended for measurement use in their present form.

**Item Numbering:** Items retain their original numbering from the scale development process (1, 2, 4, 6, 7, 8, 11, 12, 13, 14, 15, and 19). The initial pool comprised 52 items. Expert review reduced this pool first to 34 items and subsequently to the final 12 items, as described in the Methods. The original numbering is maintained in this final version to ensure consistency with Table 2 in the main manuscript, as well as to facilitate accurate cross-referencing. Item-level content validity ratios and the reasons for retention or removal of the remaining items are reported in Supplementary Table S3.

**Scoring:** Sum all 12 items (Items 1, 2, 4, 6, 7, 8, 11, 12, 13, 14, 15, and 19). Total scores range from 12 to 60, with higher scores indicating greater levels of eating disorder-specific rumination.

**Table S2.** Correlations between the ED-RRA and the EDE-Q subscales (Sample 2, n = 387)

| EDE-Q subscale | r with ED-RRA |
| --- | --- |
| Shape Concern | .75^**^ |
| Weight Concern | .72^**^ |
| Eating Concern | .67^**^ |
| Restraint | .54^**^ |
| **Note**. ^**^*p* < .001. ED-RRA = Eating Disorder-Related Rumination Assessment Scale;  EDE-Q = Eating Disorder Examination Questionnaire. | |

**Table S3.** Item-Level Expert Ratings, Content Validity Ratios (CVRs), and Reasons for Item Retention or Removal

| **Item** | **Essential ratings (n/7)** | **CVR** | **Decision** | **Rationale** |
| --- | --- | --- | --- | --- |
| Item 1 | 7 | 1.00 | Retained | Met the critical CVR threshold. Satisfied the criteria of content relevance, theoretical alignment, and clarity. |
| Item 2 | 7 | 1.00 | Retained | Met the critical CVR threshold. Satisfied the criteria of content relevance, theoretical alignment, and clarity. |
| Item 3 | 5 | 0.43 | Removed | This item reflected the same underlying content as another item, expressed in different wording. In line with expert recommendations, the most representative and clearly worded item for each concept was retained, and this item was removed for reasons of parsimony. |
| Item 4 | 7 | 1.00 | Retained | Met the critical CVR threshold. Satisfied the criteria of content relevance, theoretical alignment, and clarity. |
| Item 5 | 5 | 0.43 | Removed | Although rated as essential by five of the seven experts, following discussion of the two dissenting experts' comments and recommendations, the item's content was judged to reflect threat anticipation characteristic of anxiety (specifically, apprehension about negative outcomes associated with losing control) rather than the passive, non-solution-generating cognitive style characteristic of rumination. In addition, the item was judged unsuitable for Likert-type rating because it contained more than one judgment (i.e., a double-barreled item). To preserve a clear conceptual distinction between rumination and anxiety and to ensure clarity, the item was removed directly during the qualitative evaluation process. |
| Item 6 | 7 | 1.00 | Retained | Met the critical CVR threshold. Satisfied the criteria of content relevance, theoretical alignment, and clarity. |
| Item 7 | 7 | 1.00 | Retained | Met the critical CVR threshold. Satisfied the criteria of content relevance, theoretical alignment, and clarity. |
| Item 8 | 7 | 1.00 | Retained | Met the critical CVR threshold. Satisfied the criteria of content relevance, theoretical alignment, and clarity. |
| Item 9 | 5 | 0.43 | Removed | Although rated as essential by five of the seven experts, following discussion of the two dissenting experts' comments and recommendations, the item was judged to emphasize specific action-oriented thought content (in this item, meal planning and timing) rather than the passive, non-solution-generating cognitive style characteristic of rumination. During the qualitative evaluation process, this specific content was judged to be already comprehensively represented by the retained, more general item; where deemed necessary, the specific content was incorporated into the corresponding retained item as a parenthetical example. |
| Item 10 | 3 | −0.14 | Removed | Rated as essential by only three of the seven experts. Based on the experts' evaluations and the qualitative discussion that followed (in this item, concern that others might negatively evaluate one's weight or eating behavior in one's absence), the item was judged to reflect fear of negative evaluation rather than the conceptual content of rumination. |
| Item 11 | 7 | 1.00 | Retained | Met the critical CVR threshold. Satisfied the criteria of content relevance, theoretical alignment, and clarity. |
| Item 12 | 7 | 1.00 | Retained | Met the critical CVR threshold. Satisfied the criteria of content relevance, theoretical alignment, and clarity. |
| Item 13 | 7 | 1.00 | Retained | Met the critical CVR threshold. Satisfied the criteria of content relevance, theoretical alignment, and clarity. |
| Item 14 | 7 | 1.00 | Retained | Met the critical CVR threshold. Satisfied the criteria of content relevance, theoretical alignment, and clarity. |
| Item 15 | 7 | 1.00 | Retained | Met the critical CVR threshold. Satisfied the criteria of content relevance, theoretical alignment, and clarity. |
| Item 16 | 5 | 0.43 | Removed | Although rated as essential by five of the seven experts, following discussion of the two dissenting experts' comments and recommendations, the item was judged to emphasize a specific perfectionistic mental scenario (in this item, expectations of having greater opportunities in life) rather than the passive, repetitive, and involuntary thought patterns characteristic of rumination. During the qualitative evaluation process, this specific content was judged to be already comprehensively represented by the retained, more general items. |
| Item 17 | 6 | 0.71 | Removed | Although rated as essential by most experts, the item was judged to emphasize specific action-oriented thought content (in this item, nutritional supplements and laxatives) rather than the passive, non-solution-generating cognitive style characteristic of rumination. During the qualitative evaluation process, this specific content was judged to be already comprehensively represented by the retained, more general item; where deemed necessary, the specific content was incorporated into the corresponding retained item as a parenthetical example. |
| Item 18 | 5 | 0.43 | Removed | Although rated as essential by five of the seven experts, following discussion of the two dissenting experts' comments and recommendations, the item was judged to emphasize a specific perfectionistic mental scenario (in this item, imagined scenarios concerning romantic relationships) rather than the passive, repetitive, and involuntary thought patterns characteristic of rumination. During the qualitative evaluation process, this specific content was judged to be already comprehensively represented by the retained, more general items. |
| Item 19 | 7 | 1.00 | Retained | Met the critical CVR threshold. Satisfied the criteria of content relevance, theoretical alignment, and clarity. |
| Item 20 | 5 | 0.43 | Removed | This item reflected the same underlying content as another item, expressed in different wording. In line with expert recommendations, the most representative and clearly worded item for each concept was retained, and this item was removed for reasons of parsimony. |
| Item 21 | 5 | 0.43 | Removed | This item reflected the same underlying content as another item, expressed in different wording. In line with expert recommendations, the most representative and clearly worded item for each concept was retained, and this item was removed for reasons of parsimony. |
| Item 22 | 5 | 0.43 | Removed | This item reflected the same underlying content as another item, expressed in different wording. In line with expert recommendations, the most representative and clearly worded item for each concept was retained, and this item was removed for reasons of parsimony. |
| Item 23 | 5 | 0.43 | Removed | Although rated as essential by five of the seven experts, following discussion of the two dissenting experts' comments and recommendations, the item was judged to emphasize a specific perfectionistic mental scenario (in this item, expectations concerning social relationships) rather than the passive, repetitive, and involuntary thought patterns characteristic of rumination. During the qualitative evaluation process, this specific content was judged to be already comprehensively represented by the retained, more general items. |
| Item 24 | 5 | 0.43 | Removed | Although rated as essential by five of the seven experts, following discussion of the two dissenting experts' comments and recommendations, the item's content was judged to reflect threat anticipation characteristic of anxiety (in this item, apprehension that past negative experiences of losing control would recur) rather than the passive, non-solution-generating cognitive style characteristic of rumination. To preserve a clear conceptual distinction between rumination and anxiety, the item was removed directly during the qualitative evaluation process. |
| Item 25 | 5 | 0.43 | Removed | This item reflected the same underlying content as another item, expressed in different wording. In line with expert recommendations, the most representative and clearly worded item for each concept was retained, and this item was removed for reasons of parsimony. |
| Item 26 | 5 | 0.43 | Removed | This item reflected the same underlying content as another item, expressed in different wording. In line with expert recommendations, the most representative and clearly worded item for each concept was retained, and this item was removed for reasons of parsimony. |
| Item 27 | 5 | 0.43 | Removed | Although rated as essential by five of the seven experts, following discussion of the two dissenting experts' comments and recommendations, the item was judged to emphasize specific action-oriented thought content (in this item, exercise and calorie calculation) rather than the passive, non-solution-generating cognitive style characteristic of rumination. During the qualitative evaluation process, this specific content was judged to be already comprehensively represented by the retained, more general item; where deemed necessary, the specific content was incorporated into the corresponding retained item as a parenthetical example. |
| Item 28 | 5 | 0.43 | Removed | Although rated as essential by five of the seven experts, following discussion of the two dissenting experts' comments and recommendations, the item's content was judged to reflect threat anticipation characteristic of anxiety (in this item, apprehension about being stigmatized and subjected to prejudice) rather than the passive, non-solution-generating cognitive style characteristic of rumination. To preserve a clear conceptual distinction between rumination and anxiety, the item was removed directly during the qualitative evaluation process. |
| Item 29 | 5 | 0.43 | Removed | Although rated as essential by five of the seven experts, following discussion of the two dissenting experts' comments and recommendations, the item was judged to emphasize a specific perfectionistic mental scenario (in this item, scenarios of being a strong-willed and determined person) rather than the passive, repetitive, and involuntary thought patterns characteristic of rumination. To preserve conceptual distinctiveness, the item was judged appropriate for direct removal. |
| Item 30 | 5 | 0.43 | Removed | Although rated as essential by five of the seven experts, following discussion of the two dissenting experts' comments and recommendations, the item's content was judged to reflect threat anticipation characteristic of anxiety (in this item, apprehension about being unable to participate in valued activities) rather than the passive, non-solution-generating cognitive style characteristic of rumination. To preserve a clear conceptual distinction between rumination and anxiety, the item was removed directly during the qualitative evaluation process. |
| Item 31 | 5 | 0.43 | Removed | This item reflected the same underlying content as another item, expressed in different wording. In line with expert recommendations, the most representative and clearly worded item for each concept was retained, and this item was removed for reasons of parsimony. |
| Item 32 | 5 | 0.43 | Removed | Although rated as essential by five of the seven experts, following discussion of the two dissenting experts' comments and recommendations, the item was judged to emphasize a specific perfectionistic mental scenario (in this item, scenarios of attaining an ideal body shape and weight) rather than the passive, repetitive, and involuntary thought patterns characteristic of rumination. During the qualitative evaluation process, this specific content was judged to be already comprehensively represented by the retained, more general item; where deemed necessary, the specific content was incorporated into the corresponding retained item as a parenthetical example. |
| Item 33 | 5 | 0.43 | Removed | This item reflected the same underlying content as another item, expressed in different wording. In line with expert recommendations, the most representative and clearly worded item for each concept was retained, and this item was removed for reasons of parsimony. |
| Item 34 | 5 | 0.43 | Removed | This item reflected the same underlying content as another item, expressed in different wording. In line with expert recommendations, the most representative and clearly worded item for each concept was retained, and this item was removed for reasons of parsimony. |

**Note*.*** n = number of experts (out of N = 7) rating an item as essential. CVR = Content Validity Ratio, calculated as CVR = (n_e_ − N/2)/(N/2) (31). According to Ayre and Scally (32), the critical value for N = 7 is ≥ .99. Retention was governed by this threshold, which all 12 retained items met. The rationale column records, for each item that fell below the threshold, the content it addressed together with its relation to the retained item set.
